# Disparities in response to mRNA SARS-CoV-2 vaccines according to sex and age: A systematic review

**DOI:** 10.1016/j.nmni.2024.101551

**Published:** 2024-12-06

**Authors:** Michelle Bachmann, Nejla Gültekin, Zeno Stanga, Jan S. Fehr, Ismail I. Ülgür, Patricia Schlagenhauf

**Affiliations:** a Department of Global and Public Health, Epidemiology, Biostatistics and Prevention Institute, WHO Collaborating Centre for Travellers' Health, Centre of Competence for Military Medicine Biology, University of Zürich, Switzerland; b Swiss Armed Forces, Medical Services, Ittigen, Bern, Switzerland

## Abstract

**Background:**

The rapid development and distribution of mRNA COVID-19 vaccines has been essential in containing the SARS-CoV-2 epidemic around the globe. For ongoing and future immunization campaigns globally, there is a need to evaluate the impact of population demographics such as age and sex, on vaccine efficacy and safety.

**Methods:**

This systematic review (PROSPERO ID CRD42023328245) conducted according to PRISMA guidelines evaluates the impact of age and sex on the safety and efficacy of the mRNA COVID-19 vaccinations administrated in 15 studies that were chosen according to strict criteria. The ROBIS tool was applied to evaluate the robustness and the quality of the studies included in the review.

**Results:**

After screening, 15 studies satisfied the inclusion criteria. The results showed that the COVID-19 mRNA vaccines typically elicit robust immune responses, and that younger people have higher antibody levels. Comparing the sexes reveals that higher immunological responses were induced in females, and mild to moderate adverse effects (such as injection site discomfort, exhaustion, and headaches) were also more frequently reported in women. Despite these variations, COVID-19 vaccines have been found to be safe to use across diverse populations, which supports their extensive use in public health initiatives.

**Conclusion:**

Our results suggests that tailored vaccination may achieve maximum effectiveness and better tolerability depending on age and sex. Currently study results are rarely stratified by age and sex and this is a deficit in clinical trial publications. More research is needed to elucidate the biological mechanisms underlying the variations in vaccine responses according to age and sex.

## 1 Introduction

As of June 23, 2024, there have been 775,615,736 confirmed cases of COVID-19, including 7,051,323 deaths [1]. The infection does not discriminate among population groups; it can impact individuals irrespective of their demographic. However, it does manifest differently in terms of severity and outcome with respect to gender. Males, in particular, experience greater likelihoods of both intensive therapy unit (ITU) admission and mortality, compared to females [2].

Furthermore, in examining the factors contributing to severe COVID-19 infections, it was notably observed that elderly male patients with a high body mass index, dyspnea, and a combination of underlying diseases (such as hypertension, diabetes, cardiovascular disease, and chronic obstructive pulmonary disease) were more susceptible to developing severe forms of the illness [3]. The underlying mechanisms have yet to be determined. Nevertheless, key differences in immune responses during the disease course of SARS-CoV-2 infection in male and female patients have been revealed. At baseline, female patients exhibited a stronger T cell response, specifically in activated CD8 T cells, compared to male patients. Poor T cell responses in males were associated with future disease progression, while higher levels of innate immune cytokines were linked to worsening of COVID-19 in females. The T cell response correlated negatively with age in males but not in females. These differences suggest a benefit from a sex as well as age-dependent approaches to prognosis, prevention, care, and therapy for COVID-19 [4].

Clinical data demonstrate that there are notable distinctions between males and females in terms of vaccine-induced immune responses, adverse events, and protection across different age groups [5,6]. While the above-mentioned studies focus on clinical severity of COVID-19 by sex, the aim this systematic review is to examine the impact of sex and age on the efficacy and tolerability of mRNA vaccines against SARS-CoV-2.

## 2 Methodology

### 2.1 Search strategy

The systematic search adhered to the PRISMA (Preferred Reporting Items for Systematic Reviews and Meta-Analyses) guidelines to ensure transparency and completeness in reporting. The search was initiated on August 20, 2023, and included a comprehensive review of literature published up to July 2023. A second search was done on April 24, 2024, which included papers to cover the time period August 2023 to April 2024. Databases searched included PubMed, Ovid, Embase, Cochrane, CINAHL, Scopus, and MedRxiv. Each database was queried using a tailored approach to maximize retrieval of relevant studies, with adjustments made for the specific indexing systems and search capabilities of each database. Documentation of the search strategies was maintained through Excel and Word, facilitating a systematic review and management of the search outputs.

### 2.2 Search string

The search strategy employed a combination of keywords and MeSH (Medical Subject Headings) terms to ensure thorough retrieval of relevant studies. Keywords included: "COVID-19," "vaccine," "vaccination," "sex characteristics," "sex factors," "gender," "coronavirus," "SARS-COV-2," and "neutralizing antibodies." These keywords were combined with MeSH terms to refine the search further. ("COVID-19″ OR "SARS-CoV-2″) AND ("vaccine" OR "vaccination") AND ("sex factors" OR "gender") AND "neutralizing antibodies". This approach ensures that all relevant aspects of the study topic are covered, from the type of vaccine to the specific immune response indicators.

### 2.3 Inclusion and exclusion criteria

The studies included were those involving human subjects who received the mRNA SARS-CoV-2 vaccine and had measurements of neutralizing antibodies post-vaccination. Exclusion criteria were stringent: studies not in English, studies without clear data on vaccine type or dosing, and studies where neutralizing antibodies were not measured were excluded. Duplicates were identified and removed during the initial screening process, ensuring that each data point originated from a unique source.

### 2.4 Data extraction

The data extraction process for our systematic review was organized to ensure precision and comprehensiveness. Initially, all studies identified from the database searches were imported into Rayyan, ensuring that each study was uniquely accounted for in the analysis. The screening process involved two stages: initial and full-text review. In the initial stage, two independent reviewers, Michelle Bachmann and Patricia Schlagenhauf, evaluated the studies based on titles and abstracts to determine their relevance to the research questions, specifically focusing on those involving participants who received mRNA SARS-CoV-2 vaccines and had neutralizing antibodies measured.

Studies that passed this preliminary screening were then subjected to a full-text review to confirm their compliance with the detailed inclusion and exclusion criteria, such as specific information on vaccine type, dosage, and participant demographics. During both stages of screening, any disagreements between the reviewers were resolved through discussion. If a consensus could not be reached, a third reviewer was called upon to make a decisive judgment, ensuring the integrity and objectivity of the selection process.

Following the selection phase, data extraction was carried out using a standardized form designed to consistently capture essential information across studies. This form recorded details like study metadata, participant demographics, vaccine specifics, outcome measures related to the immune response, and any adverse events. The data were then entered into a centralized database, allowing for streamlined management and subsequent analysis. Throughout this phase, regular quality checks were performed to validate the accuracy and completeness of the data collected, comparing entries against original articles and ensuring consistency across reviewers' interpretations regarding the impact of sex and age on the response to mRNA SARS-CoV-2 vaccines.

### 2.5 Quality assessment

The quality of each selected study was assessed using the ROBIS tool, which evaluates the risk of bias in systematic reviews. This assessment covered several domains, including study eligibility criteria, identification and selection of studies, data collection and study appraisal, and synthesis and findings. Each study was independently assessed by two reviewers to ensure objectivity; discrepancies were resolved through discussion or consultation with a third reviewer. This process ensured that the conclusions drawn from the systematic review were based on high-quality and reliable evidence.

### 2.6 Data synthesis

Data synthesis involved both qualitative and quantitative approaches. Descriptive statistics were used to summarize key demographic and clinical characteristics of the study populations. Subgroup analyses were planned to assess differences in vaccine response based on sex and age groups, using chi-square tests for categorical data and t-tests for continuous data. Odds ratios (ORs) and 95 % confidence intervals (CIs) were calculated to quantify the strength of the associations observed. The narrative synthesis provided a textual explanation of the data, integrating findings from individual studies into a cohesive understanding of how sex and age influence the immune response to mRNA vaccines. This comprehensive approach allowed for a nuanced analysis of the data, ensuring that all relevant factors were considered in the interpretation of the results.

The studies included in our analysis defined vaccine response and efficacy through various measures. Primarily, these included the quantification of neutralizing antibodies as a direct immunological marker of response. T cell responses were also reported in some studies, providing additional insights into the cellular immune response. Moreover, vaccine efficacy was assessed based on clinical outcomes, such as symptomatic infection (VEi) and the reduction in hospitalization rates (VEh).

#### 2.6.1 Trial heterogeneity

Heterogeneity across the included studies was primarily assessed by examining differences in study design, population characteristics (such as age, gender, and underlying health conditions), and the methodologies used to measure immune responses (e.g., neutralizing antibodies, T cell responses, clinical outcomes). We also considered geographical location and the type of vaccine used as potential sources of variability.

While we did not conduct a formal meta-analysis due to the diverse nature of these factors, we acknowledged this heterogeneity qualitatively in the Results section. Further analysis, such as subgroup analyses or meta-regression, was not feasible given the limited number of studies and the diversity of measurements used. Additionally, differences between the mRNA vaccines were not considered in our analysis. This was due to the fact that studies, who included more than one mRNA vaccine did not always clearly describe the exact number of participants who received each specific mRNA vaccine [13]. Moreover, it was noted in some cases that no significant differences in efficacy were observed between the vaccines [14,20]

## 3 Results

**Fig. 1:**
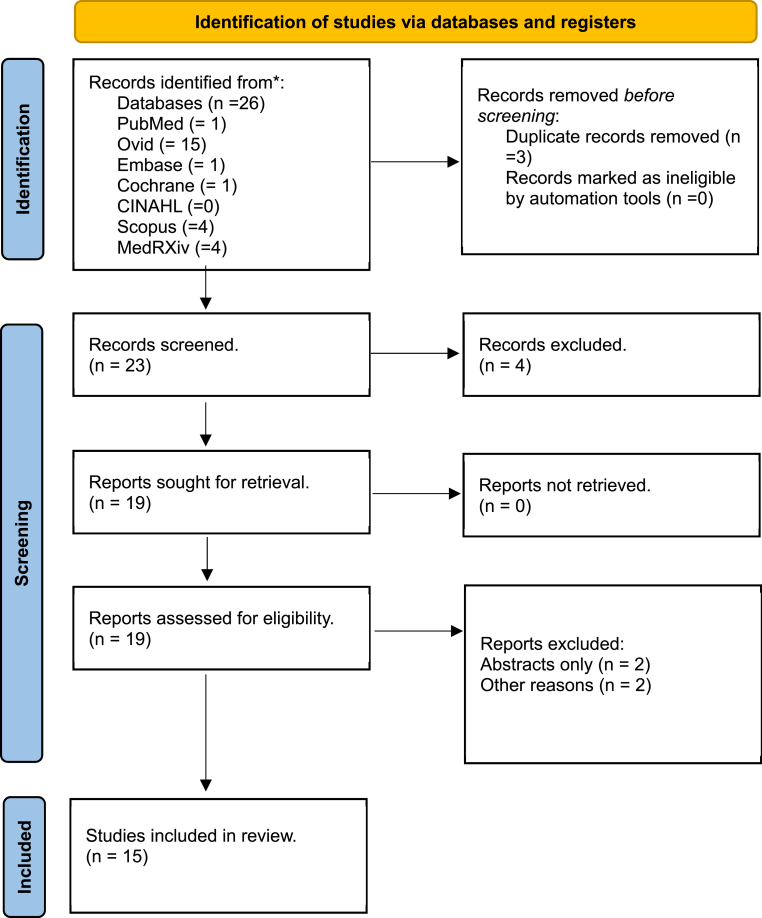
PRISMA flow diagram for the Systematic review *Disparities in response to mRNA SARS-CoV-2 vaccines according to sex and age*.

**Fig. 2:**
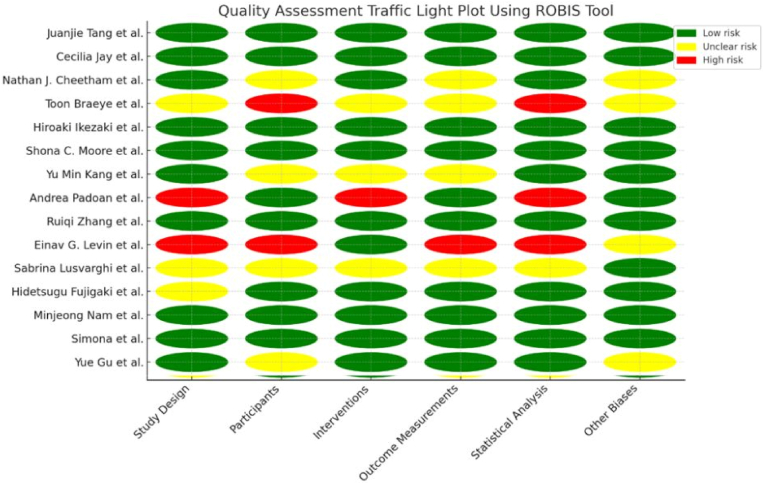
Traffic light graph of the quality assessment and risk of bias of included studies, Green represents the low risk, yellow represents the moderate risk and red represents the high risk.

The total of 26 studies were retrieved after the comprehensive search across different databases. After the removal of duplicates and applying the exclusion criteria a total of 15 studies*,* encompassing a total of 1,449,905 participants, finally met the inclusion criteria and were included in this systematic review. The detailed flow diagram of inclusion and screening process of studies is given in Fig. 1. The quality assessment of all the studies were analyzed using ROBIS tool. The detailed traffic light graph of the quality assessment of included studies is given in Fig. 2. Across the 15 studies included, the overall efficacy of COVID-19 vaccines was demonstrated through significant increases in antibody levels and neutralizing responses post-vaccination. Younger individuals consistently exhibited higher antibody responses compared to older individuals. For instance, Jay et al. [7] and Zhang et al. [8] found that adolescents and younger healthcare workers had higher IgG and anti-S levels than their older counterparts. This trend underscores the robust immune response in younger populations.

Sex differences were also evident, with females generally exhibiting higher antibody levels and stronger immune responses than males. Anticoli et al. [9] and Kang et al. [10] reported that female healthcare workers and younger women had significantly higher anti-S/RBD antibody concentrations than males. However, females also experienced more frequent adverse events and a more rapid decline in antibody levels over time, as highlighted by studies such as Gu et al. [11] and Nam et al. [12].

The safety profiles of the vaccines were consistent across studies, with most adverse events being mild to moderate in severity. Common adverse events included injection site pain, fatigue, and headache. Younger individuals and females reported higher rates of adverse events than older individuals and males. However, the overall severity of adverse events did not differ significantly between age and sex groups. Booster vaccinations effectively enhanced and maintained antibody levels, particularly in older populations, mitigating some of the age-related decline in vaccine efficacy [7,10,[13], [14], [15]].

The studies demonstrate that mRNA COVID-19 vaccines elicit strong immune responses, particularly in younger and female individuals. The increase in antibody levels and neutralizing responses post-vaccination indicates that the vaccines provide substantial protection against SARS-CoV-2. The observed higher efficacy in younger populations suggests that age significantly influences vaccine response [11,12,[16], [17], [18], [19]].

### 3.1 Outcome by age, sex and age & sex

This section synthesizes data from the included studies to analyze how different age groups responded to COVID-19 vaccines in terms of efficacy, correlates of immunity, levels of protection and safety, as summarized in Table 1, Table 2, Table 3. Tang et al. [20], which involved 69 naive and 17 convalescent adults, found no significant age-dependent association with neutralizing titers, indicating that age did not significantly impact the vaccine-induced antibody response. Jay et al. [7] targeted adolescents aged 12–16 years and adults aged 32–52 years, finding that total IgG responses to SARS-CoV-2 Spike antigens were significantly higher among vaccinated adolescents compared to adults, with infection-naive male adolescents showing higher antibody responses than females (62,270 vs. 36,951, p = 0.008). In Cheetham et al. [14], which included 9361 individuals, older age was associated with a lower incidence of high antibody levels post-vaccination, though a third vaccination dose increased antibody levels for almost all individuals, mitigating some of the age-related decline. Braeye et al. [13] analyzed 1,433,135 persons for vaccine effectiveness against symptomatic infection (VEi) and 662,220 symptomatic infected persons for vaccine effectiveness against hospitalization (VEh). The study found that younger individuals had higher vaccine effectiveness during the Delta-dominant fourth wave, which reversed during the Omicron-dominant fifth wave.

**Table 1:** Vaccine efficacy by age.

Study	Age Group	Type of Vaccine	Number of Participants	Correlates of immunity, levels of protection	Outcome
[20]	All Ages	mRNA-1273 (Moderna) or BNT162b2 (Pfizer BioNTech)	69 naive and 17 convalescent adults	NAb, significant increase after second dose in both groups	No significant age-dependent association with neutralizing titers observed
[7]	Adolescents (12–16) vs Adults (32–52)	BNT162b2 (Pfizer BioNTech)	34 adolescents aged between 12 and 16 and 589 adults aged 32-52	Anti-S/RBD IgG, NAb, and T-cell responses, significant increases in anti-S/RBD IgG post first dose, significant increases in NAb post second dose, T-cell responses increased post second dose	Higher IgG responses in adolescents compared to adults
[14]	Older Adults	AZD1222 (Oxford/AstraZeneca) BNT162b2 (Pfizer BioNTech)	9361	Anti-S IgG, peak titers after third dose, decline after first and second dose but stable after third dose.	Older individuals had lower immune responses to vaccination
[17]	60+	BNT162b2 (Pfizer BioNTech)	49 participants (Analysis-1). 373 participants (Analysis-2)	Anti-S IgG, peak titers after second dose with a decline over 6 months	Lower anti-spike IgG levels compared to younger age groups
[19]	All Ages	BNT162b2 (Pfizer BioNTech) and AZD1222 (Oxford/AstraZeneca)	684	Anti-S/RBD IgG, NAb, T cell responses, decrease in NAb 6 months post second dose but increase after third dose. Significant IgG response after third dose, stronger T-cell response after second dose.	IgG responses decreased with age
[10]	<55 vs 55+	BNT162b2 (Pfizer BioNTech) and AZD1222 (Oxford/AstraZeneca)	309	NAb and Anti-S IgG, significant increase of NAb and anti-S after second dose.	Higher antibody levels in younger age groups post first dose
[15]	65+ vs 18-45	BNT162b2 (Pfizer BioNTech)	4868	NAb, peak titers after second dose with a decline over 6 months.	Significant decline in neutralizing antibodies in older age groups
[18]	All Ages	BNT162b2 (Pfizer BioNTech)	39	NAb, peak titers after third dose	No association between age and neutralizing titers
[16]	Elderly	BNT162b2 (Pfizer BioNTech)	219	RBD-IgG, IgM, and Ig, significant increase after second dose	Lower RBD-IgG levels in elderly males
[12]	All Ages	BNT162b2 (Pfizer BioNTech and AZD1222 (Oxford/AstraZeneca) and mRNA-1273 (Moderna)	573	RBD IgG and NAb, peak titers after second dose	Decrease in RBD Ab and NAb with aging
[9]	20-44 vs 56+	BNT162b2 (Pfizer BioNTech)	521	Anti-S/RBD IgG, highest response t16, significant drop after 5 months	Higher anti-S levels in younger HCWs
[11]	<40	BNT162b2 (Pfizer BioNTech)	168	NAb, significant increase after second dose.	Stronger initial neutralizing response but significant decrease over time
[13]	All Ages	BNT162b2 (Pfizer BioNTech) and AZD1222 (Oxford/AstraZeneca) and Ad2OV2.S (Johnson & Johnson), and mRNA-1273 (Moderna)	1,433,135	Vaccine effectiveness against infection (VEi) and vaccine effectiveness against hospitalization (VEh), decrease in VEi and VEh after first and second dose with slower protection decline after second dose.	Varying VE with age and virus variant
[21]	All Ages	BNT162b2 (Pfizer BioNTech)	174	NAb, significant increase t28 after first dose	No correlation with age
[8]	All Ages	BNT162b2 (Pfizer BioNTech)	189	virus microneutralization (vMN) titers, Seroprotection in 96.7 % after second dose.	Higher virus microneutralization (vMN) titer in females; no correlation with age

**Table 2:** Vaccine efficacy by sex.

Study	Sex	Type of Vaccine	Correlates of immunity, level of protection	Outcome
[20]	Male vs Female	mRNA-1273 (Moderna) or BNT162b2 (Pfizer BioNTech)	NAb, significant increase after second dose in both groups	No significant differences in neutralizing titers; higher antibody affinities in naive males
[7]	Male vs Female (Adolescents)	BNT162b2 (Pfizer BioNTech)	Anti-S/RBD IgG, NAb, and T-cell responses, significant increases in anti-S/RBD IgG post first dose, significant increases in NAb post second dose, T-cell responses increased post second dose	Higher antibody responses in males compared to females among infection-naïve adolescents
[14]	Male vs Female	AZD1222 (Oxford/AstraZeneca) BNT162b2 (Pfizer BioNTech)	Anti-S IgG, peak titers after third dose, decline after first and second dose but stable after third dose.	Males showed stronger antibody responses among adolescents
[17]	Male vs Female	BNT162b2 (Pfizer BioNTech)	Anti-S IgG, peak titers after second dose with a decline over 6 months	No significant association with sex
[19]	Male vs Female	BNT162b2 (Pfizer BioNTech) and AZD1222 (Oxford/AstraZeneca)	Anti-S/RBD IgG, NAb, T cell responses, decrease in NAb 6 months post second dose but increase after third dose. Significant IgG response after third dose, stronger T-cell response after second dose.	No effect on IgG responses; lower T cell responses in males
[10]	Male vs Female	BNT162b2 (Pfizer BioNTech) and AZD1222 (Oxford/AstraZeneca)	NAb and Anti-S IgG, significant increase of NAb and anti-S after second dose.	Higher titers in females post first dose
[15]	Male vs Female	BNT162b2 (Pfizer BioNTech)	NAb, peak titers after second dose with a decline over 6 months.	Lower titers in older men compared to women
[18]	Male vs Female	BNT162b2 (Pfizer BioNTech)	NAb, peak titers after third dose	No association with sex
[16]	Male vs Female	BNT162b2 (Pfizer BioNTech)	RBD-IgG, IgM, and Ig, significant increase after second dose	Lower RBD-IgG levels in males after second vaccination
[12]	Male vs Female	BNT162b2 (Pfizer BioNTech and AZD1222 (Oxford/AstraZeneca) and mRNA-1273 (Moderna)	RBD IgG and NAb, peak titers after second dose	Higher RBD Ab, NAb, and IGR levels in females
[9]	Male vs Female	BNT162b2 (Pfizer BioNTech)	Anti-S/RBD IgG, highest response t16, significant drop after 5 months	Higher anti-S/RBD antibody levels in females; more rapid decline in females
[11]	Male vs Female	BNT162b2 (Pfizer BioNTech)	NAb, significant increase after second dose.	Higher antibody levels and stronger responses in females
[13]	Male vs Female	BNT162b2 (Pfizer BioNTech) and AZD1222 (Oxford/AstraZeneca) and Ad2OV2.S (Johnson & Johnson), and mRNA-1273 (Moderna)	Vaccine effectiveness against infection (VEi) and vaccine effectiveness against hospitalization (VEh), decrease in VEi and VEh after first and second dose with slower protection decline after second dose.	Lower VE in males against Delta post-primary vaccination
[21]	Male vs Female	BNT162b2 (Pfizer BioNTech)	NAb, significant increase t28 after first dose	No significant differences in antibody response
[8]	Male vs Female	BNT162b2 (Pfizer BioNTech)	virus microneutralization (vMN) titers, Seroprotection in 96.7 % after second dose.	Women showed higher levels of neutralizing antibodies; more frequent AEs in women

**Table 3:** Vaccine efficacy by age and sex.

Study	Age Group	Sex	Type of Vaccine	Correlates of immunity, level of protection	Outcome
[20]	All Ages	Both	mRNA-1273 (Moderna) or BNT162b2 (Pfizer BioNTech)	NAb, significant increase after second dose in both groups	No significant differences by age or sex
[7]	Adolescents vs Adults	Both	BNT162b2 (Pfizer BioNTech)	Anti-S/RBD IgG, NAb, and T-cell responses, significant increases in anti-S/RBD IgG post first dose, significant increases in NAb post second dose, T-cell responses increased post second dose	Higher IgG in adolescents; sex difference only in adolescents (males with higher antibody responses)
[14]	Older Adults	Both	AZD1222 (Oxford/AstraZeneca) BNT162b2 (Pfizer BioNTech)	Anti-S IgG, peak titers after third dose, decline after first and second dose but stable after third dose.	Older individuals had lower immune responses; sex difference in adolescents (males with higher antibody responses)
[17]	60+	Both	BNT162b2 (Pfizer BioNTech)	Anti-S IgG, peak titers after second dose with a decline over 6 months	Lower anti-spike IgG levels in older adults; no significant association with sex
[19]	All Ages	Both	BNT162b2 (Pfizer BioNTech) and AZD1222 (Oxford/AstraZeneca)	Anti-S/RBD IgG, NAb, T cell responses, decrease in NAb 6 months post second dose but increase after third dose. Significant IgG response after third dose, stronger T-cell response after second dose.	IgG responses decreased with age; lower T cell responses in males
[10]	<55 vs 55+	Both	BNT162b2 (Pfizer BioNTech) and AZD1222 (Oxford/AstraZeneca)	NAb and Anti-S IgG, significant increase of NAb and anti-S after second dose.	Higher antibody levels in younger groups; higher titers in females post first dose
[15]	65+ vs 18-45	Both	BNT162b2 (Pfizer BioNTech)	NAb, peak titers after second dose with a decline over 6 months.	Significant decline in older age groups; lower titers in older men compared to women
[18]	All Ages	Both	BNT162b2 (Pfizer BioNTech)	NAb, peak titers after third dose	No association with age or sex
[16]	Elderly	Both	BNT162b2 (Pfizer BioNTech)	RBD-IgG, IgM, and Ig, significant increase after second dose	Lower RBD-IgG levels in elderly males; no significant association in females
[12]	All Ages	Both	BNT162b2 (Pfizer BioNTech and AZD1222 (Oxford/AstraZeneca) and mRNA-1273 (Moderna)	RBD IgG and NAb, peak titers after second dose	Decrease in RBD Ab and NAb with aging; higher levels in females
[9]	20-44 vs 56+	Both	BNT162b2 (Pfizer BioNTech)	Anti-S/RBD IgG, highest response t16, significant drop after 5 months	Higher anti-S levels in younger HCWs; more rapid decline in females
[11]	<40	Both	BNT162b2 (Pfizer BioNTech)	NAb, significant increase after second dose.	Higher initial neutralizing response in younger; higher levels in females
[13]	All Ages	Both	BNT162b2 (Pfizer BioNTech) and AZD1222 (Oxford/AstraZeneca) and Ad2OV2.S (Johnson & Johnson), and mRNA-1273 (Moderna)	Vaccine effectiveness against infection (VEi) and vaccine effectiveness against hospitalization (VEh), decrease in VEi and VEh after first and second dose with slower protection decline after second dose.	Varying VE with age and virus variant; lower VE in males post-primary vaccination
[21]	All Ages	Both	BNT162b2 (Pfizer BioNTech)	NAb, significant increase t28 after first dose	No correlation with age; no significant differences with sex
[8]	All Ages	Both	BNT162b2 (Pfizer BioNTech)	virus microneutralization (vMN) titers, Seroprotection in 96.7 % after second dose.	Higher vMN titer in females; no correlation with age

Hiroaki Ikezaki et al. [17] focused on healthcare workers and found that older age groups, specifically those aged 60 years and older, had substantially lower anti-Spike IgG levels six months post-vaccination, with the odds ratio of maintaining anti-Spike IgG levels above 2150 AU/ml being less than 0.3 for individuals aged 60 and above. Moore et al. [19] examined long-term vaccine-induced and hybrid immunity in 684 healthcare workers and found that IgG responses decreased with age, indicating an age-related decline in vaccine-induced immunity, with male sex associated with lower T cell responses in the AZD1222 (Oxford/AstraZeneca) group. Kang et al. [10] included 309 participants and found that younger individuals in the BNT162b2 (Pfizer/BioNTech) group displayed significantly higher anti-S antibody levels after the first dose compared to older individuals, although no significant age-related differences were observed after the second dose. Padoan et al. [21], in a study with 174 participants receiving the BNT162b2 vaccine, found no significant correlation between age and neutralizing antibody titers measured by different assays.

Zhang et al. [8] included 189 participants and found that recipients of the BNT162b2 vaccine had higher neutralizing antibody titers on day 56, with younger healthcare workers (20–44 years) developing higher anti-S levels than those aged over 56 years. Levin et al. [15], in a study with 4868 participants, reported that participants aged 65 and older exhibited lower levels and a more substantial decline in neutralizing antibodies compared to those aged 18 to less than 45, with men over 45 showing significantly lower antibody titers compared to younger men, and a 46 % decrease in neutralizing antibody concentrations in men aged 65 and older compared to women in the same age group. Lusvarghi et al. [18] included 39 participants and found no association between age and neutralizing titers against the Omicron BA.1 (Pfizer/BioNTech) variant after two doses of the BNT162b2 vaccine.

Fujigaki et al. [16] studied 219 participants and found that elderly males had a lower RBD-IgG antibody-producing ability than their younger counterparts, with RBD-IgG levels significantly lower in males than females after the second vaccination dose. Nam et al. [12] examined 573 participants and found that age significantly influenced cellular immune responses, with older individuals showing a decrease in receptor-binding domain antibodies (RBD Ab) and neutralizing antibodies (NAb) but an increase in interferon gamma release (IGR) levels. Anticoli et al. [9], with 521 participants, found that younger healthcare workers (20–44 years) developed higher anti-S/RBD antibody levels than those in the older age groups, with females having higher anti-S/RBD antibody levels than males, and a more abrupt decline in response over time observed in women compared to men. Gu et al. [11] included 168 participants and found that individuals below 40 years old had a stronger initial neutralizing response, but antibody levels decreased significantly in all age groups by six months post-vaccination, with females exhibiting higher antibody levels and stronger neutralizing responses than males.

In another study by Jay et al. [7] involving 34 adolescents and 589 adults, previously infected adolescents showed robust neutralizing responses after one dose, while infection-naive adolescents required two doses, with adolescents showing significantly higher IgG responses than adults aged 32–52, and no significant difference in vaccine-induced immunity between males and females. Overall, these studies consistently show that younger individuals exhibit higher antibody responses and greater vaccine effectiveness compared to older individuals, with adolescents and young adults often having more robust immune responses, while older age groups show a decline in antibody levels and overall vaccine-induced immunity. Booster vaccinations are particularly effective in enhancing antibody levels across all age groups, helping to offset some of the age-related decline in vaccine efficacy.

### 3.2 Safety and adverse events

**Fig. 3:**
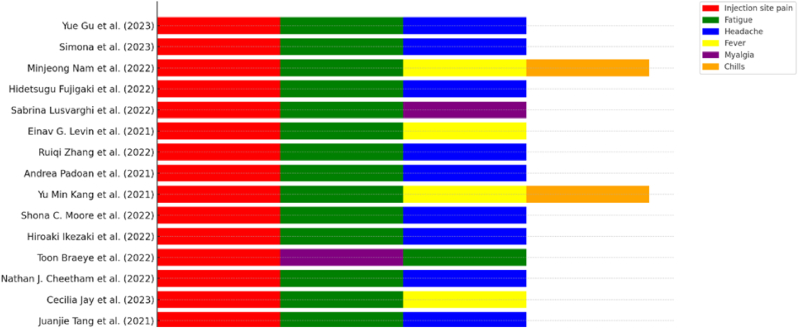
The adverse events related to the vaccination among different age groups reported in the included studies.

Regarding safety, the vaccines were well-tolerated across different age and sex groups, with reported adverse events primarily mild to moderate and resolving within a few days (Fig. 3). While females and younger individuals experienced higher rates of adverse events, these did not significantly impact the overall safety profile of the vaccines, which was consistent across studies, supporting their widespread use in diverse populations. According to Tang et al. [20], adverse events were generally mild to moderate, with injection site pain, fatigue, and headache being the most common. No significant differences in adverse events were observed between males and females, though naive individuals reported slightly more adverse events compared to convalescent individuals. Similarly, Jay et al. [7] noted that adolescents reported more frequent adverse events than adults, particularly injection site pain, fatigue, and headache, with no significant differences between males and females in terms of frequency or severity. Cheetham et al. [14] found that while adverse events were common, they were mostly mild to moderate, with older individuals reporting fewer adverse events compared to younger ones, and no significant sex differences overall, though younger males reported slightly higher rates of systemic symptoms like fever and chills. Braeye et al. [13] reported that adverse events, mostly injection site reactions, fatigue, and headache, were generally mild and resolved within a few days, with higher rates in younger individuals and females. Ikezaki et al. [17] observed similar trends, noting that common adverse events such as injection site pain, fatigue, and myalgia were more frequently reported by younger individuals, with no significant sex differences. Moore et al. [19] highlighted that younger participants, particularly females in the AZD1222 vaccine group, reported more frequent adverse events, predominantly injection site pain and systemic symptoms like fatigue and fever. Kang et al. [10] found that women in both the ChAdOx1 (Oxford/AstraZeneca) and BNT162b2 groups reported higher rates of adverse events compared to men, with younger participants reporting more adverse events overall. Padoan et al. [21] noted that adverse events, including injection site pain, fatigue, and headache, were mild to moderate, with no significant differences between age groups or sexes. Zhang et al. [8] observed that younger healthcare workers (20–44 years) reported higher rates of adverse events compared to older workers, with women reporting more frequent adverse events than men, including injection site pain and systemic symptoms like fever and chills. Levin et al. [15] found that older participants reported fewer adverse events compared to younger ones, with older males reporting fewer adverse events than older females. Common adverse events included injection site reactions, fatigue, and headache. Lusvarghi et al. [18] reported no significant association between age or sex and the frequency of adverse events, which were mostly mild to moderate, including injection site pain, fatigue, and headache. Fujigaki et al. [16] observed that elderly males reported fewer adverse events compared to younger participants and females, with common adverse events being injection site pain, myalgia, and fatigue. Nam et al. [12] found that females, particularly in younger age groups, exhibited higher rates of adverse events compared to males, with common adverse events being injection site pain, fatigue, and headache. Older participants reported fewer adverse events compared to younger participants. Anticoli et al. [9] noted that female healthcare workers, especially younger females (20–44 years), reported more frequent adverse events, primarily injection site pain, fatigue, and systemic symptoms like fever. Gu et al. [11] found that females exhibited higher rates of adverse events compared to males, with younger females reporting the highest rates. Common adverse events included injection site pain, fatigue, and headache, with older participants reporting fewer adverse events. In another study by Jay et al. [7], previously infected adolescents reported fewer adverse events compared to infection-naive adolescents, who required two doses to achieve similar antibody responses. Overall, adverse events were mild to moderate, with no significant differences between males and females. Summarizing the safety profiles, the studies consistently showed that the COVID-19 vaccines were well-tolerated, with most adverse events being mild to moderate and resolving within a few days. Injection site pain, fatigue, and headache were the most commonly reported adverse events. Younger individuals and females generally reported higher rates of adverse events compared to older individuals and males, but the severity was not significantly different across age and sex groups. Booster doses were well-tolerated and did not significantly increase the incidence of adverse events, highlighting the importance of ongoing monitoring to ensure optimal vaccination strategies and public health outcomes.

## 4 Discussion

Age significantly impacts the immune response to COVID-19 vaccines, with younger individuals showing higher antibody levels and better overall vaccine efficacy. Sex differences are evident, with females generally exhibiting stronger immune responses but also experiencing more frequent adverse events. The combination of age and sex further elucidates variations, as younger females showed the highest antibody responses and highest incidence of adverse events. It is acknowledged that older age groups tend to exhibit a decline in antibody levels, a phenomenon potentially influenced by immunosenescence, which impairs immune function with age. While it is possible that younger individuals may have experienced natural exposures that could boost their antibody levels, contributing to their higher response rates, this could have impacted the observed results.

The scarcity of available data, stratified by both age and sex, from vaccine producers and other studies complicates comprehensive comparisons. Despite these limitations, large sample sizes in studies provide robust data [13,14].

The search for studies examining the impact of age and sex on COVID-19 vaccine efficacy and safety faced significant challenges. Firstly, the literature on this specific intersection is limited. Despite the abundance of research on COVID-19 vaccines, few studies simultaneously consider both age and sex. This gap necessitated a more extensive search across various databases, yet the results remained sparse. Additionally, direct inquiries to vaccine producers yielded no substantial data, as they reported having no specific information related to sex and age variations. This deficit in clinical data reporting highlights a critical barrier in obtaining comprehensive data, further limiting the scope of available research.

The primary limitation of this systematic review is the insufficient number of studies that directly compare vaccine efficacy and safety across different sexes and ages. Many studies stratify efficacy and tolerability by sex or by age, but few cross stratify by sex and age.

This scarcity results in a reliance on indirect comparisons and fragmented data. Moreover, vaccine efficacy was assessed using multiple metrics, including neutralizing antibody levels, symptomatic infection rates, and reductions in hospitalization, aiming to provide a comprehensive evaluation. However, assumptions about the relationship between antibody levels and vaccine effectiveness introduce potential biases. Without these data, significant gaps remain, particularly in understanding long-term effects and specific subgroup responses. The heterogeneity in study designs, populations, and outcome measures further complicates direct comparisons, potentially introducing bias and reducing generalizability and leaving significant gaps, particularly in understanding long-term effects and subgroup responses.

Despite the limitations, this study boasts several strengths. Firstly, the search was comprehensive, covering multiple databases and employing rigorous inclusion criteria. And studies were tested for bias using the ROBIS tool. This thorough approach ensures that the most relevant studies were considered. Secondly, the review includes controversial and timely research on vaccines developed rapidly during the pandemic. This context highlights the dynamic nature of vaccine research and the importance of continuous monitoring. Thirdly, the large sample sizes in the included studies enhance the reliability of the findings. These studies collectively offer a substantial evidence base for evaluating vaccine efficacy and safety across diverse populations.

A notable finding across studies is the higher incidence of adverse events in women compared to men. Women consistently report more frequent and severe side effects post-vaccination. Common adverse events include injection site pain, fatigue, and headaches, which are typically mild to moderate in severity and resolve within a few days. While the higher frequency of these events in women may point to underlying biological mechanisms, it is also possible that differences in reporting and health-seeking behavior between men and women contributed to this observation. Although none of the studies explicitly addressed this aspect, the potential influence of such variations cannot be discounted. Hormonal differences, particularly estrogen levels, may play a significant role in modulating immune responses and adverse events. Studies have suggested that estrogen enhances immune function, potentially leading to both stronger immune responses and higher rates of adverse reactions [7,9,17].

The hormonal state is a critical factor influencing vaccine responses, particularly in women. Estrogen and progesterone, which vary across the menstrual cycle, pregnancy, and menopause, can significantly affect immune function. Estrogen, in particular, has been shown to enhance the immune response, leading to higher antibody levels and stronger reactions to vaccines [[7], [8], [9],^11^,^12^,^16^]. This heightened immune response can contribute to the increased incidence of adverse events observed in women [9]. Understanding these hormonal influences is crucial for developing tailored vaccination strategies that optimize efficacy while minimizing adverse effects. Further research is needed to elucidate the precise mechanisms through which hormones impact vaccine responses and to develop guidelines for managing these effects in clinical practice [7,8,12].

While sex differences in immunogenicity and adverse events can be hypothesized, the expedited decay of antibody titers remains less understood, with potential contributions from factors like age and immune system regulation. Further investigation into this complex issue is necessary to guide future vaccination strategies [11,13,14,16].

The findings from this review have significant implications for public health strategies and future research. Prioritizing vaccination in younger populations, who exhibit stronger immune responses, could expedite the achievement of herd immunity. Additionally, recognizing the higher efficacy and adverse event rates in females necessitates tailored communication strategies to maintain vaccine confidence and adherence. Transparent public health messaging that addresses potential side effects, particularly in women and younger individuals, is essential to sustain public trust and promote widespread vaccination [7,8,12,14,18,20,21].

Future research should focus on several key areas. Firstly, long-term studies are needed to assess the durability of vaccine-induced immunity, particularly in older populations who experience a more rapid decline in antibody levels [11,13,14,16]. Investigating the effects of booster doses and their role in sustaining immunity will be critical for ongoing vaccination efforts [11,13,14,16,18,20,21]. Secondly, more research is required to understand the biological mechanisms underlying sex differences in immune responses and adverse events [9]. This knowledge can inform the development of gender-specific vaccination strategies and improve overall vaccine safety and efficacy [11,13,16].

## 5 Conclusion

In conclusion, this systematic review highlights the complex interplay between age, sex, and mRNA COVID-19 vaccine responses. While younger individuals and females generally exhibit higher antibody levels and stronger immune responses, they also report more frequent adverse events. These findings underscore the need for targeted public health strategies and continued research to optimize vaccine efficacy and safety across diverse populations. It is worth mentioning, that vaccine manufacturers have the capability to conduct extensive studies; however, they have been reticent in publishing the resultant data stratified by both age and sex. Nevertheless, the rapid development and widespread administration of COVID-19 vaccines provide a unique opportunity to study these variations and improve our understanding of vaccine responses.

## CRediT authorship contribution statement

**Michelle Bachmann:** Writing – original draft. **Nejla Gültekin:** Project administration. **Zeno Stanga:** Writing – review & editing, Project administration. **Jan S. Fehr:** Project administration. **Ismail I. Ülgür:** Writing – review & editing, Project administration. **Patricia Schlagenhauf:** Supervision, Conceptualization, Analyses, Supervision, Writing and review of manuscript.

## Funding

The authors acknowledge the support of the Centre of Competence for Military and Disaster Medicine, Swiss Armed Forces, Bern, Switzerland.

## Declaration of competing interest

The authors declare that they have no known competing financial interests or personal relationships that could have appeared to influence the work reported in this paper.
